# Endothelial -targeted CD39 is protective in a mouse model of global forebrain ischaemia

**DOI:** 10.1186/s12974-025-03394-7

**Published:** 2025-04-21

**Authors:** Natasha Ting Lee, Ioanna Savvidou, Carly Selan, David K. Wright, Robert Brkljaca, Joanne S.J. Chia, Ilaria Calvello, Daphne D.D. Craenmehr, Pia Larsson, Volga Tarlac, Amy Vuong, Irena Carmichael, Xiaowei Wang, Karlheinz Peter, Simon C. Robson, Harshal H. Nandurkar, Maithili Sashindranath

**Affiliations:** 1 Present Address: Australian Centre for Blood Diseases, School of Translational Medicine, Alfred Hospital, Monash University, Melbourne, VIC 3004 Australia; 2 Department of Neuroscience, School of Translational Medicine, Monash University, Melbourne, VIC 3004 Australia; 3 Monash Biomedical Imaging, Monash University, Clayton, VIC 3168 Australia; 4 Center for Inflammation Research, Department of Anesthesia, Critical Care & Pain Medicine, Division of Gastroenterology, Department of Medicine, Beth Israel Deaconess Medical Center, Harvard Medical School, Boston, MA USA; 5 Atherothrombosis and Vascular Biology Laboratory, Baker Heart and Diabetes Institute, Melbourne, VIC Australia; 6 Department of Cardiometabolic Health, University of Melbourne, Melbourne, VIC Australia; 7 Molecular Imaging and Theranostics Laboratory, Baker Heart and Diabetes Institute, Melbourne, VIC Australia; 8 Present Address: Monash Micro Imaging-ARA, Sub-Faculty of Translational Medicine and Public Health, Monash University, 89 Commercial Road, Melbourne, VIC 3004 Australia

**Keywords:** Ischaemia, Dual carotid artery ligation, Global hypoxia, CD39, VCAM-1

## Abstract

Global ischemic brain injury occurs after cardiac arrest or prolonged hypotensive episodes following surgery or trauma. It causes significant neurological deficits even after successful re-establishment of blood flow. It is the primary cause of death in 68% of inpatient and 23% of out-of-hospital cardiac arrest cases, but there are currently no treatments. Endothelial activation and dysfunction impairing small vessel blood flow is the cause of brain damage. Purinergic signaling is an endogenous molecular pathway, where CD39 and CD73 catabolize extracellular adenosine triphosphate (eATP) to adenosine. After ischemia, eATP is released, triggering thrombosis and inflammation. In contrast, adenosine is anti-thrombotic, protects against oxidative stress, and suppresses the immune response. Our group developed a bifunctional compound – *anti-VCAM-CD39* that targets the dysregulated endothelium and promotes adenosine generation at the infarct site, localising the antithrombotic and anti-inflammatory effects of CD39. We investigated whether *anti-VCAM-CD39* could improve outcome in a murine model of global ischaemia caused by dual carotid artery ligation (DCAL). Test drugs *anti-VCAM-CD39* and controls were given 3 h after 30 min ischaemia. Assessments at 24 h included neurological function, infarct volume, perfusion, and albumin extravasation to assess blood-brain barrier (BBB) permeability. We showed that there was an overall improvement in neurological deficit in anti-VCAM-CD39-treated mice after DCAL. MRI revealed that these mice had significantly smaller infarcts and reduced apoptotic activity on the side of permanent occlusion, compared to saline treated mice. There was reduced albumin extravasation in treated mice after DCAL, suggesting *anti-VCAM-CD39* conferred neuroprotection in the brain through preservation of BBB permeability. In vitro findings confirmed that anti-VCAM-CD39-mediated adenosine production protected against hypoxia-induced endothelial cell death. *anti-VCAM-CD39* is a novel therapeutic that can promote neuroprotection, reduce tissue damage and inflammation after hypoxic brain injury in mice. These findings suggest that *anti-VCAM-CD39* could be a new avenue of cardiac arrest therapy and could potentially be used in other cerebrovascular diseases where endothelial dysfunction is a constant underlying pathology.

**Supplementary Information:**

The online version contains supplementary material available at 10.1186/s12974-025-03394-7.

## Introduction

Hypoxic–ischemic encephalopathy or global hypoxic ischaemic brain injury (HIBI) is a consequence of global cerebral ischaemia due to cardiac arrest or after prolonged hypotensive episodes, such as hanging, strangulation or drowning [1]. It is a leading cause of mortality and is associated with significant long-term neurological disability in survivors [2]. The brain has a high metabolic need and is entirely dependent on a continuous supply of oxygen and glucose from the blood for proper functioning. HIBI can only be managed by establishing adequate oxygen delivery, thereby limiting secondary brain injury but there is currently no conclusive treatment [3]. Therefore, there is an unmet clinical need for new therapeutics.

As little as 5 min of hypoxic insult can cause widespread brain damage [4], including neuronal cell death, blood-brain barrier (BBB) permeability and tissue necrosis [5]. Two pathological processes mediate brain injury after HIBI. The first phase is the initial injury due to oxygen and glucose deprivation when blood flow is disrupted. The second phase is the ischaemia reperfusion injury (IRI) that follows re-establishment of blood flow after resuscitation following cardiac arrest [6].

IRI leads to endothelial cell (EC) injury and release of extracellular adenosine triphosphate (eATP). eATP signals via purinergic P2 X_1 − 7_ receptors on macrophages and T-cells to boost pro-inflammatory cytokines (tumour necrosis factor (TNF-α), IL-6 and IL-8, IL-1β and IL-18) production [7, 8]. EC activation during IRI leads to increased expression of vascular cell adhesion molecule-1 (VCAM-1) [9] while hypoxia enhances transendothelial migration of T-cells into the brain due to increased intercellular adhesion molecule-1 (ICAM-1) which is mediated by Hypoxia-inducible factor-1 (HIF-1)-α [10]. Leukocytes use VCAM-1 as one of the receptors to adhere to ECs and infiltrate tissues, amplifying inflammation. P- and E-selectins and von Willebrand factor (vWF) are also released, leading to a local prothrombotic environment [11].

CD39 is an ecto-nucleoside triphosphate diphosphohydrolase (NTPDase) expressed by ECs and is the dominant regulator of purinergic signalling in the vasculature [12]. eATP and eADP form substrates for CD39 enzymatic activity that converts a pro-inflammatory and pro-thrombotic vascular-EC environment to an anti-inflammatory and anti-thrombotic phenotype by the generation of adenosine. Adenosine production catalysed by CD39/CD73 is critical in protecting tissue against hypoxic and ischaemic insults [13]; CD39 reduces intravascular thrombosis [14] and CD39^–/–^ mice have larger infarct volumes after stroke [15]. We developed a new molecule, *anti-VCAM-CD39* which consists of recombinant CD39 fused to a single chain fragment variable of VCAM-1 antibody. A unique construct that confers the anti-inflammatory and antithrombotic effect of CD39 to areas of endothelial dysfunction characterised by upregulation of VCAM-1, we recently showed that *anti-VCAM-CD39* was protective in a mouse model of ischaemic stroke, administered at a dose that was well below the threshold of haemostatic defect [16]. Given the overlapping pathophysiology of ischaemic stroke and global HIBI, we considered whether targeted delivery of CD39 to the activated endothelium would improve neurological outcome in a model of global forebrain IRI that we previously developed [17].

## Results

### VCAM-1 is upregulated within the infarcted areas of the brain and anti-VCAM-CD39 binds to activated endothelial cells

IRI induces upregulation of endothelial VCAM-1 within the ischemic zone at 3 h post-DCAL (suppl Fig. 1), in accordance with previous findings [18]. Selective binding of our construct anti-VCAM-CD39 to activated endothelial cells was confirmed in vitro. Murine endothelial cells were stimulated with pro-inflammatory TNF-α overnight, fluorescently labelled anti-VCAM-CD39 was added followed by anti-VCAM-1 antibody. We were able to show co-localisation of anti-VCAM-CD39 with VCAM-1 on the surface of stimulated endothelial cells (Supplementary Fig. 1.2A). We also showed that non-targeted CD39 does not bind to VCAM-1 using an ELISA (Supplementary Fig. 1.2 B). Next, we used intravital microscopy to confirm binding of anti-VCAM-CD39 in vivo, using the same labelled construct. Figure 1.3 shows that anti-VCAM-CD39 localises to the vascular bed labelled with CD31 and VCAM after laser induced endothelial injury (Supplementary Fig. 1.3 and supplementary video 1).

### *anti-VCAM-CD39* ameliorates brain damage in mice subjected to DCAL while reducing blood brain barrier permeability and limiting endothelial activation

**Fig. 1:**
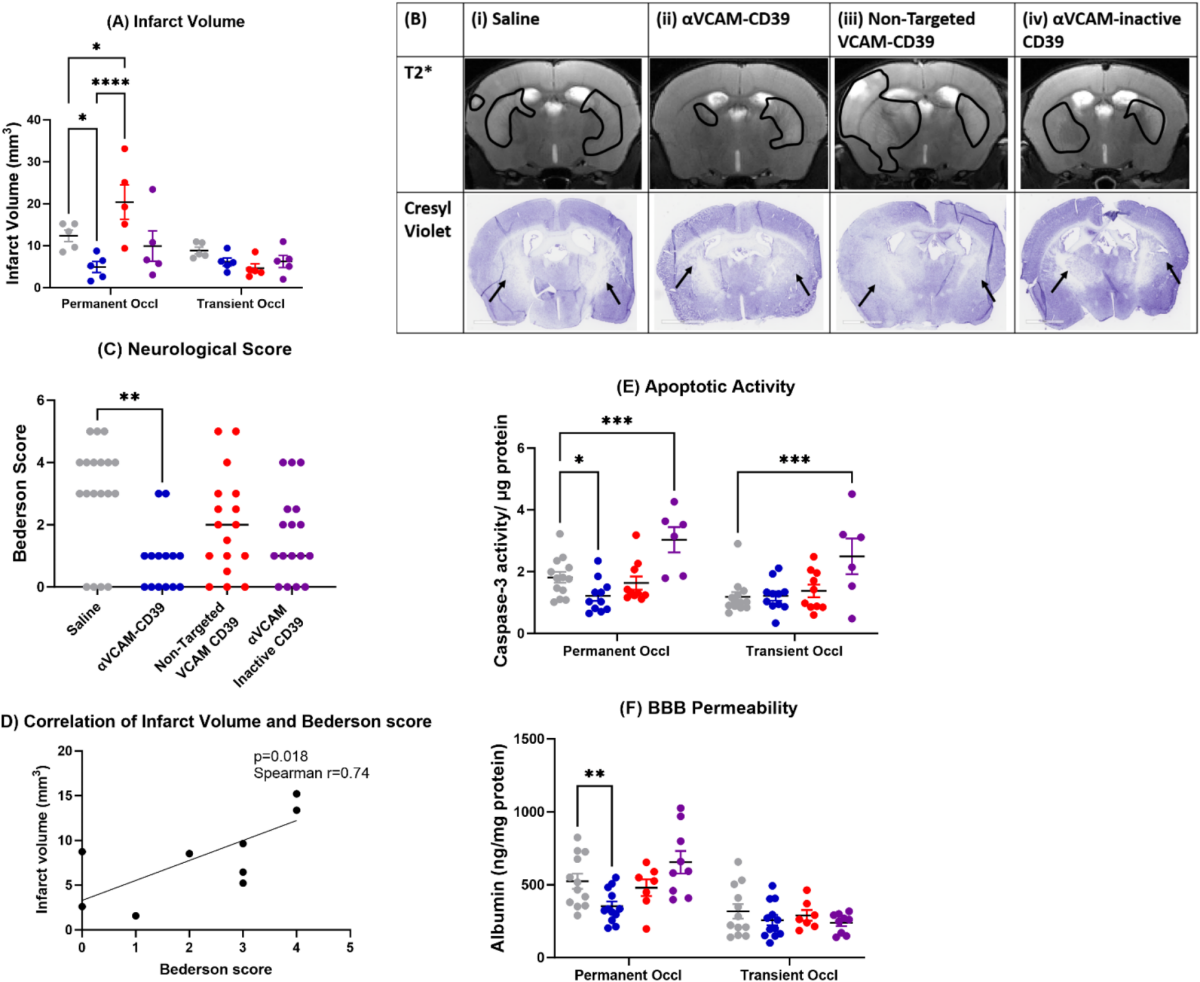
anti-VCAM-CD39 is effective in mitigating brain damage following global forebrain hypoxia. (**A**) MRI-based DWI-quantified infarct volumes (mm^3^) of anti-VCAM-CD39 treated mouse brains 24 h post-DCAL showed significantly lower infarct volumes on the side of permanent occlusion compared to saline, non-targeted CD39 and anti-VCAM-inactive CD39 controls. (Data is Mean ± SEM; Saline: *n* = 5, anti-VCAM-CD39: *n* = 5; *p < 0.05; one-way ANOVA with Sidak’s post‐hoc analysis). (**B**) MR T2* imaging and Cresyl Violet histological staining of mouse brains post-DCAL after treatment with (i) saline, (ii) anti-VCAM-CD39, (iii) non-targeted CD39, (iv) anti-VCAM-inactive CD39 (*n* = 4–5) distinctly show the infarcted area indicated by delineation, and this injury and corresponding absence of Cresyl violet positive cells in the same region, indicated by the arrows, confirming cell death. (**C**) anti-VCAM-CD39 significantly reduced neurological score compared to saline, and also had lower scores compared to drug control groups (Saline: *n* = 19, anti-VCAM-CD39: *n* = 14–17; Bars indicate median value, **p < 0.01, ***p < 0.001; Kruskal Wallis test with Dunnett’s post‐hoc analysis).(**D**) Infarct volume correlates with Bederson scores (*n* = 10). (**E**) Caspase activity 24-hours post-DCAL is significantly increased in the site of permanent occlusion, and anti-VCAM-CD39 was able to significantly decrease apoptotic activity. (Data is Mean ± SEM; Saline: *n* = 13, anti-VCAM-CD39: *n* = 6–11; *p < 0.05, ***p < 0.001, ****p < 0.0001; two-way ANOVA with Uncorrected Fisher’s LSD). (**F**) BBB permeability measured by albumin extravasation in the brain parenchyma was significantly increased on the side of permanent occlusion post-DCAL, and anti-VCAM-CD39 treatment was effective in reducing albumin extravasation (Data is Mean ± SEM; Saline: *n* = 12, anti-VCAM-CD39: *n* = 12; *p < 0.05; one‐way ANOVA with Sidak’s post‐hoc analysis)

We found that after 0.125 mg/kg of *anti-VCAM-CD39* treatment, there was a significant decrease in the total infarct volume assessed by MRI-based T2* analysis (Fig. 1A), with distinct bilateral lesions evidenced by the representative MR images (Fig. 1B and Supplementary Fig. 3) that were smaller in the *anti-VCAM-CD39* treated group. Treatment with non-targeted CD39 did not significantly reduce infarct while there was a protective trend seen with VCAM blockade, when compared to saline. In fact, the non-targeted CD39 yielded a significantly larger infarct on the side of permanent occlusion, when compared to the saline, *anti-VCAM-CD39*, and *anti-VCAM-inactive CD39* treated groups. (*p* = 0.0219,* p < 0.0001, p = 0.0001* respectively) (Fig. 1A and B). Corresponding Cresyl Violet-stained sections taken from the same brains clearly delineated the infarcts and absence of Cresyl violet positive cells in the same region, confirming cell death (Fig. 1B). After anti-VCAM-CD39 treatment, there was less cell damage, with intact and round Nissl bodies observed in both hemispheres (Supplementary Fig. 2.2).

As previously shown [18], saline-treated mice post-DCAL had significantly impaired neurological function compared to sham (*p = 0.001*). These mice had a median Bederson score of 4, but mice treated with *anti-VCAM-CD39* treatment had a median of 1 (Fig. 1C). Non-targeted CD39 and *anti-VCAM-inactive CD39* were unable to reduce neurological deficit, confirming the synergistic effect seen of both CD39 activity and VCAM-blockade with treatment with *anti-VCAM-CD39.* Importantly, there was a strong positive correlation between infarct volume and Bederson scores (Spearman *r* = 0.74, *p* = 0.018; *n* = 10; Fig. 1D).

Apoptotic activity is normally increased due to ischaemic injury and corresponds to lesion volume as an indicator of cell death, which we have also previously demonstrated [18]. Apoptotic activity was significantly decreased in the permanent occlusion side of the brain 24 h after treatment with *anti-VCAM-CD39* (Fig. 1E) although it remains to be determined which cells are being rescued from apoptosis. On the side of transient occlusion, there seemed to be lower apoptotic activity overall, and similar levels of cell death in both saline and *anti-VCAM-CD39* treated groups. Interestingly, on both sides of the brain, apoptotic activity seemed to be unchanged after non-targeted CD39 when compared to saline treated animals. However, after *anti-VCAM-inactive CD39* treatment, apoptotic activity was significantly increased from saline on both sides of the brain (*p < 0.0001*), suggesting that VCAM-blockade alone was not adequate for protection against apoptosis.

We found a significant reduction in extravasated albumin post *anti-VCAM-CD39* treatment as compared to saline (Fig. 1F), suggesting a reduction in BBB permeability. Non-targeted CD39 was not able to reduce albumin extravasation, while the trend was to increase BBB permeability with *anti-VCAM- inactive CD39* (*p = 0.052*).

### *anti-VCAM-CD39* significantly reduced inflammatory cytokines and reduced hypoxic-ischaemic injury post-DCAL

**Fig. 2:**
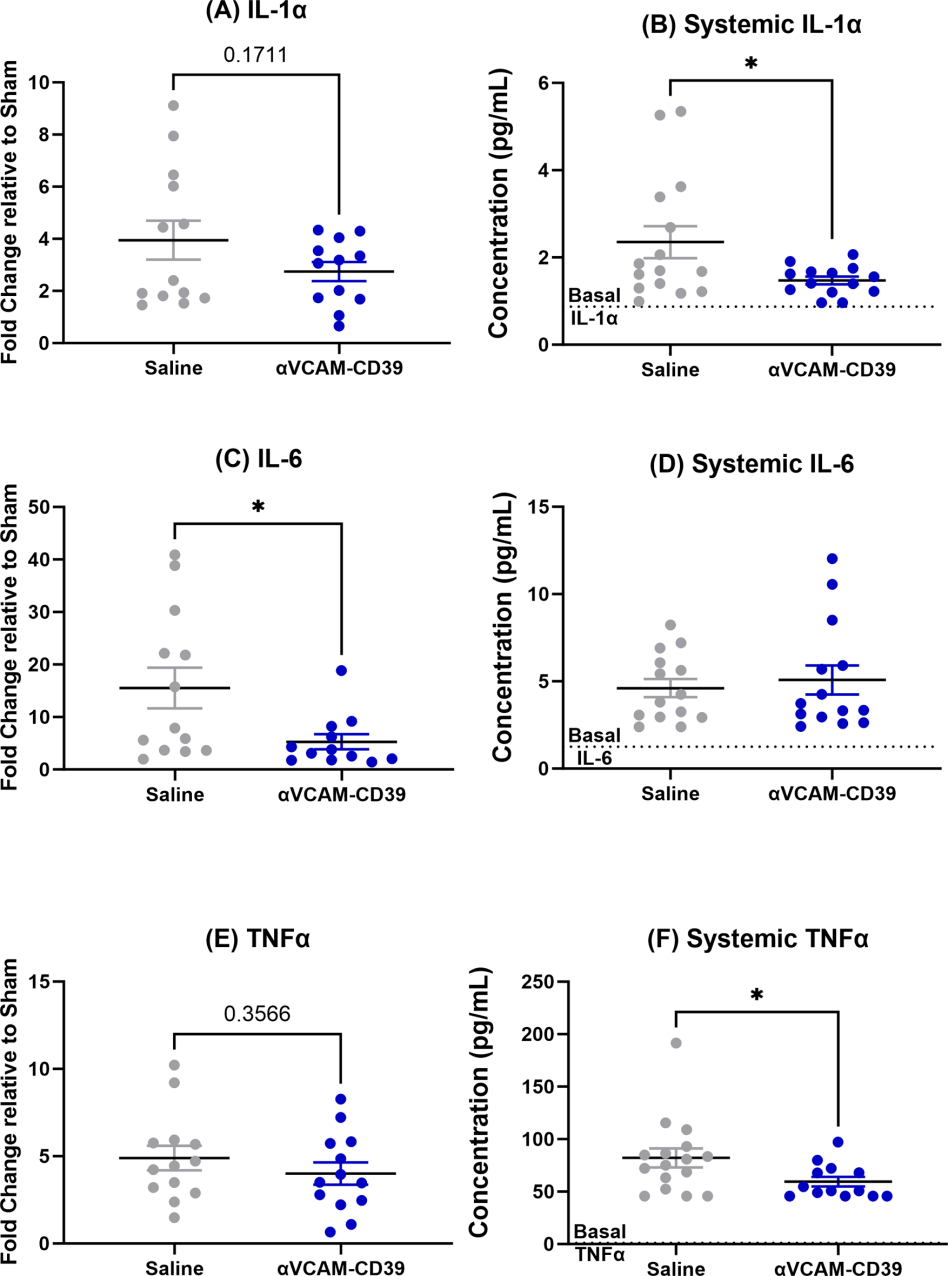
*anti-VCAM-CD39* reduces in situ gene expression and circulating plasma levels of inflammatory cytokines post-DCAL. (**A**) Gene expression of IL-1α was not significantly affected while (**B**) systemic levels of IL-1α was significantly reduced. (**C**) While gene expression of IL-6 was significantly reduced and (**D**) systemic levels were not affected. Finally, similar to IL-1α, (**E**) gene expression of TNFα were not changed while its (**F**) systemic levels were significantly reduced after *anti-VCAM-CD39* treatment. (Data is Mean ± SEM; Saline: *n* = 13–15, *anti-VCAM-CD39*: *n* = 12–15, Unpaired t-test)

We further analysed both the circulating plasma levels and tissue expression of cytokines that have been previously shown to be significantly increased following global forebrain ischaemia with DCAL [18] to gain an insight into the protective mechanism of *anti-VCAM-CD39*. Interestingly, it seems that it was the circulating plasma levels and not the gene expression of inflammatory cytokines systemic 1L1α (Fig. 2A and B) and TNFα (Fig. 2E and F) that were significantly decreased post treatment, while IL-6 (Fig. 2C and D) was only significantly decreased within the brain tissue.

**Fig. 3:**
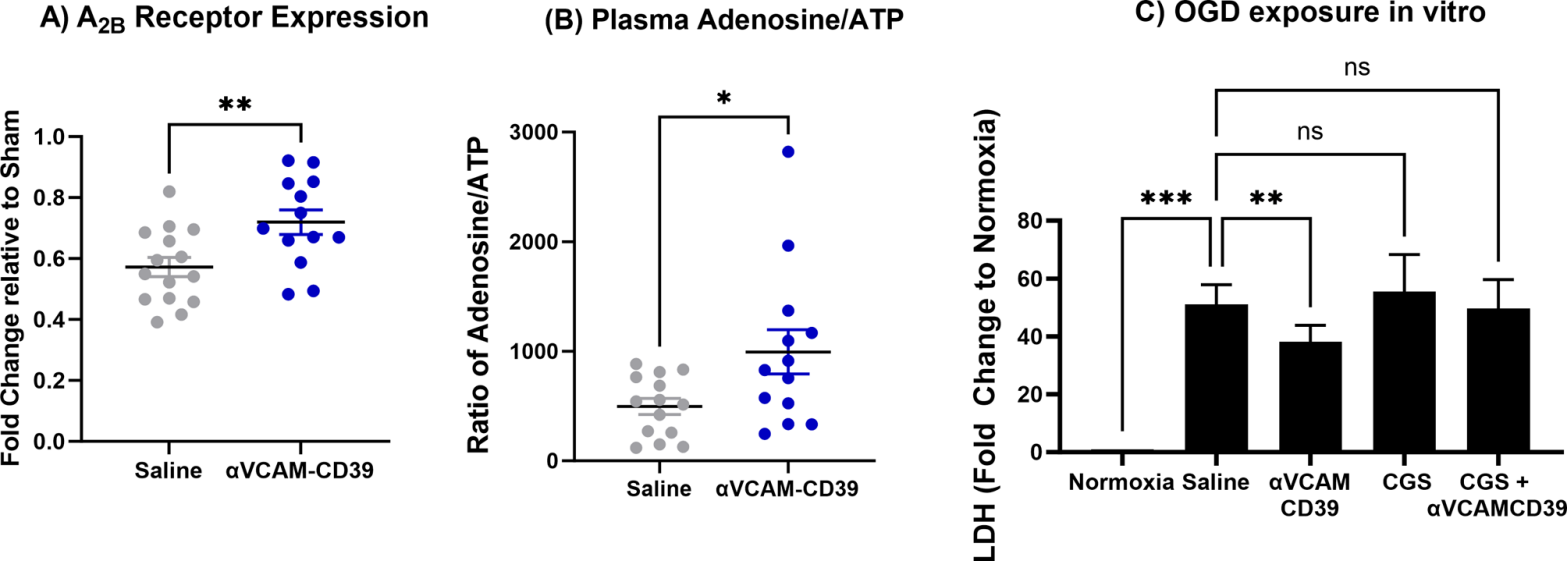
Gene expression of A_2B_ receptors were significantly changed after *anti-VCAM-CD39* treatment, and antagonism of the adenosine receptors removed the protective effect of anti-VCAM-CD39 in vitro, confirming that the protective mechanism of *anti-VCAM-CD39* is through the generation of adenosine. Expression of (**A**) A_2B_ receptor expression was found to be significantly upregulated after treatment with *anti-VCAM-CD39.* (Data is Mean ± SEM; Saline: *n* = 6–15, *anti-VCAM-CD39*: *n* = 6–13; *p < 0.05, **p < 0.01; Unpaired t-test). (**B**) Systemic Adenosine and ATP concentration (expressed as a ratio) in plasma was found to be significantly increased after *anti-VCAM-CD39* treatment (Data is Mean ± SEM; Saline: *n* = 14, *anti-VCAM-CD39*: *n* = 13; *p < 0.05; Unpaired t-test). (**C**) Analysis of LDH levels In Vitro post-OGD determined that *anti-VCAM-CD39* was able to significantly reduce necrotic cell death, but with co-treatment of CGS-15,943, this protective effect ceases (Data is Mean ± SEM; Saline: *n* = 13, *anti-VCAM-CD39*: *n* = 13; CGS-treated: *n* = 5; **p < 0.01, ***p < 0.001; Mixed-effects analysis)

We also analysed the gene expression of adenosine receptors (Fig. 3B) to understand their involvement. We found when *anti-VCAM-CD39* was administered post-DCAL, there was a significant increase in gene expression of A_2B_ receptor (Fig. 3A) suggesting that adenosine generation was likely involved in the overall improvement of I/R injury found post-DCAL. No change in A_2A_ receptor expression was detected (not shown).

To confirm the protective role of adenosine, we investigated the ratio of adenosine and ATP in the plasma 24-hours post treatment. The increase in the plasma adenosine/ATP ratio confirms that *αVCAM-CD39* created a more protective, adenosine-rich milieu (Fig. 3B). Additionally, the finding that increased adenosine/ATP ratio correlated with reduction in Bederson scores further affirms the strong protective potential of adenosine signalling (*p* = 0.0229; Spearman *r*=-0.5325; *n* = 18 (Supplementary Fig. 3)).

To further confirm whether *anti-VCAM-CD39* confers protection through adenosine signalling, we subjected mouse brain endothelial cells to oxygen glucose deprivation (OGD) and treated them with CGS-15943, a specific adenosine receptor antagonist. We confirmed that OGD caused significant necrotic cell death (Fig. 3C), and treatment with *anti-VCAM-CD39* was capable of significantly reducing LDH levels. When we treated the endothelial cells with CGS-15943 in addition to *anti-VCAM-CD39*, we observed a loss of the protective effect seen with *anti-VCAM-CD39* treatment, suggesting that blocking adenosine signalling could limit the protective effect of *anti-VCAM-CD39.*

**Fig. 4:**
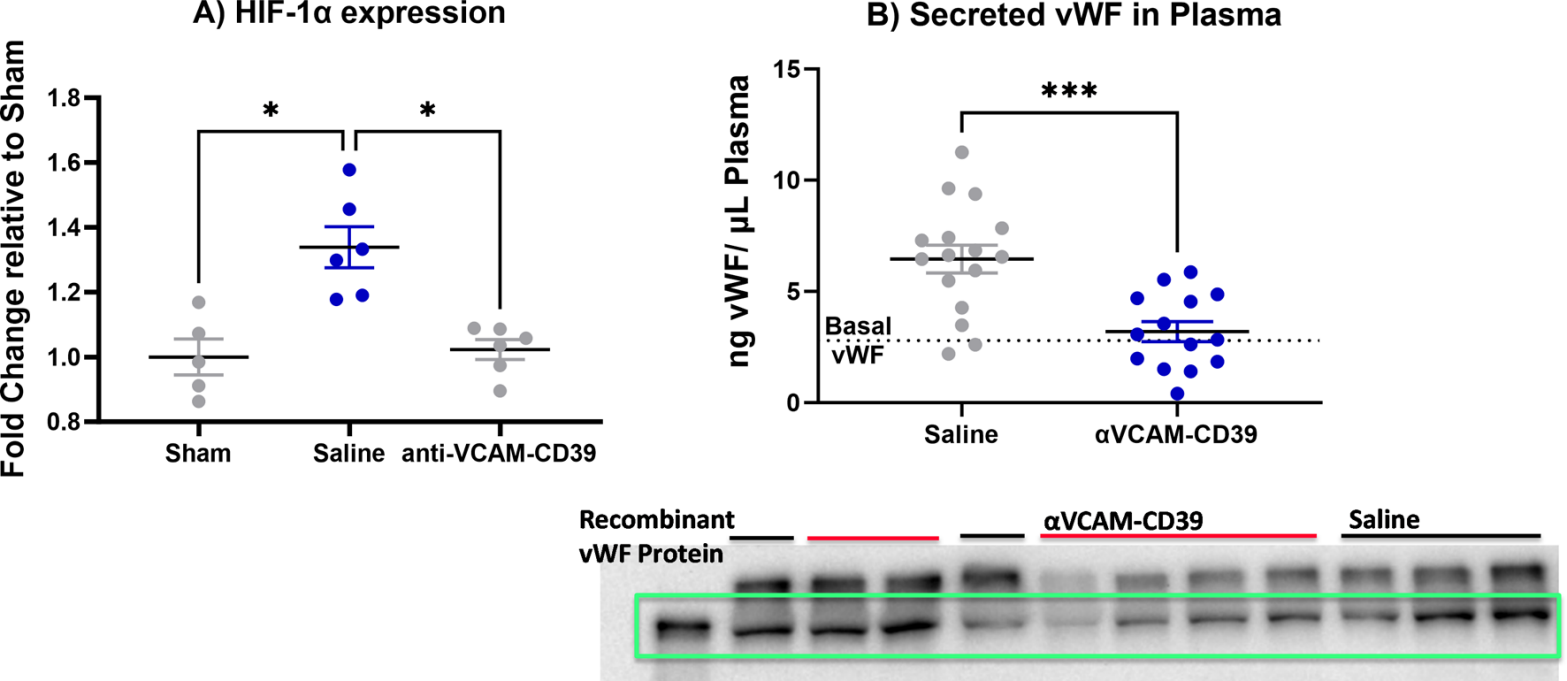
anti-VCAM-CD39 reduces hypoxic-ischaemic injury in the brain (**A**) HIF-1α was significantly reduced after anti-VCAM-CD39 treatment and (**B**) Circulating vWF in plasma was found to be significantly reduced after anti-VCAM-CD39 treatment (Data is Mean ± SEM; Saline: *n* = 16, Treated: *n* = 14; ***p < 0.001; unpaired t-test). Complete blot visible in Supplementary Fig. 4. (**C**) Endothelial cells immunolocalised with CD31 reveals that endothelial expression of ICAM-1 in the perilesional zone (indicated by arrows) is downregulated following anti-VCAM-CD39 treatment; higher magnification images are provided for the region marked with yellow squares

That anti-VCAM-CD39 resulted in a significant decrease in the gene expression of HIF-1α (Fig. 4A) while also minimising thromboinflammation, detected by secretion of vWF into the plasma suggests that the mechanism of action of this agent is via reducing hypoxic-ischaemic injury in the brain. Indeed, although there was uniform ICAM-1 expression that colocalises with CD31 + endothelial cells in the brain of sham animals (Fig. 4), the distribution of CD31 + ICAM-1 + cells in the saline treated group was more intense around the ischaemic lesion. The reduction in ICAM-1 staining intensity in the perilesional portion of the brain after anti-VCAM-CD39 is also consistent with reduced endothelial activation (Fig. 4), further supporting the role of this construct in restoring BBB integrity.

## Discussion

Global hypoxic ischaemic brain injury (HIBI) is a condition that induces widespread brain damage due to oligemia and IRI [19]. We previously characterised a novel model of global HIBI [18] and confirmed that following injury, there were bilateral lesions in the forebrain, as well as increased BBB permeability; these are common indicators of IRI and oligemia in similar global ischaemia models [20–22]. VCAM-1 biodistribution in the brain correlates strongly with BBB permeability, since it is a marker of endothelial activation [23] and therefore, targeting CD39 to VCAM-1 ensured the therapeutic delivery of CD39 to the injured brain parenchyma. That VCAM-1 antibodies demonstrate a > 10-fold increase brain uptake and brain: blood ratio following TNF-α stimulation [24] and our own in vivo data showing the selective upregulation of VCAM-1 in the brain after global ischaemia, affirms our rationale to target VCAM-1 for selective delivery of CD39 to areas of brain injury and inflammation.

Given the demonstrated efficacy of *anti-VCAM-CD39* in an ischemic stroke model [16], we hypothesised that *anti-VCAM-CD39* would be able to protect the brain against global HIBI due to the known effects of suppression of inflammatory eATP and generation of protective adenosine in improving hypoxia induced endothelial damage [25]. The neuroprotective effect of *anti-VCAM-CD39* in reducing infarct volume reflected our data from the ischaemic stroke model [16]. This effect was not observed with control constructs (non-targeted CD39 and *anti-VCAM-inactive CD39)* demonstrating the targeted effect of low-dose CD39 for neuroprotection. The increase in plasma adenosine/eATP ratio confirms the dual hydrolytic effects of *anti-VCAM-CD39* on both ATP and ADP. The functional effect of a reduction in infarct volume was evident with a reduction in Bederson score following *anti-VCAM-CD39* treatment. While reduced caspase-3 activity in the permanently occluded side of the brain following *anti-VCAM-CD39* correlates with a decrease in lesion volume in this cohort, the anti-VCAM component appeared to promote apoptotic activity. That there was no corresponding increase in infarct volume suggests that neuronal death was not increased in this cohort relative to vehicle administration. Our findings align with those of Justicia et al. 2006, who found that blocking VCAM-1 in an ischaemic mouse model alters the composition of the infiltrating leukocytes (by reducing monocyte infiltration and increasing neutrophils and lymphocytes), which might not necessarily be beneficial for the outcome of stroke. Additionally, a recent paper investigated the potential effects of Natalizumab, a recombinant antibody targeted the integrin α4ß1 (VLA4) that prevents its interaction with VCAM, in the brains of patients with relapse remitting multiple sclerosis. The report shows that although the drug blocked the transmigration of immune cells into the CNS as expected, it did not change macrophage and microglia infiltration within active demyelinating white matter biopsy lesions. This provides clinically relevant data to show that VCAM-1 blockade alone is not sufficient to diminish neuroinflammation [26].

The endothelium is a critical component of the BBB and endothelial permeability increases significantly in response to ischaemia and hypoxia, and extracellular adenosine is known to reduce hypoxia-associated vascular leakage [27]. We have previously confirmed that anti-VCAM-CD39 binds to VCAM-1 and minimises transendothelial migration of leukocytes [16] and also provide in vivo intravital imaging data showing anti-VCAM-CD39 localised to the vascular bed after laser induced endothelial injury. Although a recent manuscript reported that inhibition of VCAM1 activation protects against BBB breakdown in a mouse model of chronic cerebral hypoxia, in our experiments VCAM-1 blockade alone did not reduce albumin extravasation [23]. Reduced capacity for adenosine generation (due to gene CD39 or CD73 gene deletion) results in significant increases in vascular leakage following exposure to ambient hypoxia. CD39-mediated enzymatic processing of ATP represents the major pathway of adenosine formation during oxygen supply imbalances and endothelial cells primarily express A_2A_ and A_2B_ receptor subtypes. Adenosine-mediated activation of A_2B_ receptors has been found to decrease endothelial paracellular permeability during leukocyte transmigration [13]. Eltzschig and coworkers have previously shown that adenosine activation of A_2B_ receptor protects against leukocyte induced endothelial barrier disruption by increasing intracellular cyclic AMP formation and a consequent activation of protein kinase A [25]. Protein kinase A induces phosphorylation of vasodilator-stimulated phosphoprotein (VASP), which strengthens tight junction proteins and restores endothelial barrier function [28].

Release of vWF is a feature of endothelial activation [29] and consistent with the prothrombotic effects of IRI. Plasma vWF increased post-DCAL when compared to sham, while treatment with *anti-VCAM-CD39* significantly reduced plasma vWF, while ICAM-1 staining was reduced within the vicinity of the ischaemic zone, thereby confirming reduced endothelial activation and injury. This coincided with significant BBB protection, evidenced by a reduction in albumin extravasation after *anti-VCAM-CD39* treatment.

Endothelial cell activation because of hypoxia creates a proinflammatory and procoagulant state driven by the rapid induction of NF-κB, a transcription factor, that promotes expression of proinflammatory cytokines including TNF-α, IL-1, and IL-6. Accordingly, Berti et al. have shown that gene expression of these cytokines in the brain is increased within 3 h of ischaemia and persists for over 24 h [30]. The combined antithrombotic and anti-inflammatory effects of *anti-VCAM-CD39* in the cerebrovascular circulation reduced hypoxia in the brain, which is likely reflected by a reduction in HIF-1α expression and correlated with a reduction in IL-6 expression and a reduction in plasma levels of IL-1α and TNFα. Both cytokines regulate HIF-1α activity in brain endothelial cells promoting hypoxia and inflammation as well as increasing BBB permeability and angiogenesis [31]. In the brain, HIF-1α promotes TNFα-induced neuronal apoptosis as well as caspase-3 mediated pericyte apoptosis [32]. Here we showed an increase in gene expression of the A_2B_ receptor together with a correlative reduction in HIF-1α expression in the brains of mice after *anti-VCAM-CD39* treatment.

A_2B_ receptor signalling is also known to provide neuroprotection and reduce pro-inflammatory cytokines TNF-α, IL-1, and IL-6 from endothelial cells [33], which aligns with our data. Dettori et al. showed that chronic administration of an A_2B_ receptor agonist was able to significantly reduce infarct volume, BBB disruption and neurologic deficit after focal ischaemia in rats by preventing astrocytes loss in the striatum and reducing microglial activation [34]. A_2B_R-knockout mice have increased baseline levels of TNF-α and IL-6 that are further upregulated following LPS challenge. These mice also have high levels of the adhesion molecules E-selectin, P-selectin, and ICAM-1 in the vasculature which corresponded with heightened rolling of leukocytes and increased adhesion to the vessel [35]. In support of this, LPS treatment in CD39 deficient mice results in increased microglial activation, and concomitant activation of IL-6 signalling pathways [36].

In this work we have not identified which other cell types were involved in the reduction of HIF-1α, IL1$\:{\upalpha\:},\:$IL6 and TNFα gene expression, or which cells are being rescued by anti-VCAM-CD39, and further immunolocalization experiments are needed to identify the contribution of astrocytes, pericytes and microglia which are all known to have a role in neuroprotection after ischaemia [37]. We also note that due to limited resources and the large number of experiments, it was unfeasible for us to use both male and female mice and future experiments should include similar analyses in female mice.

**Fig. 5:**
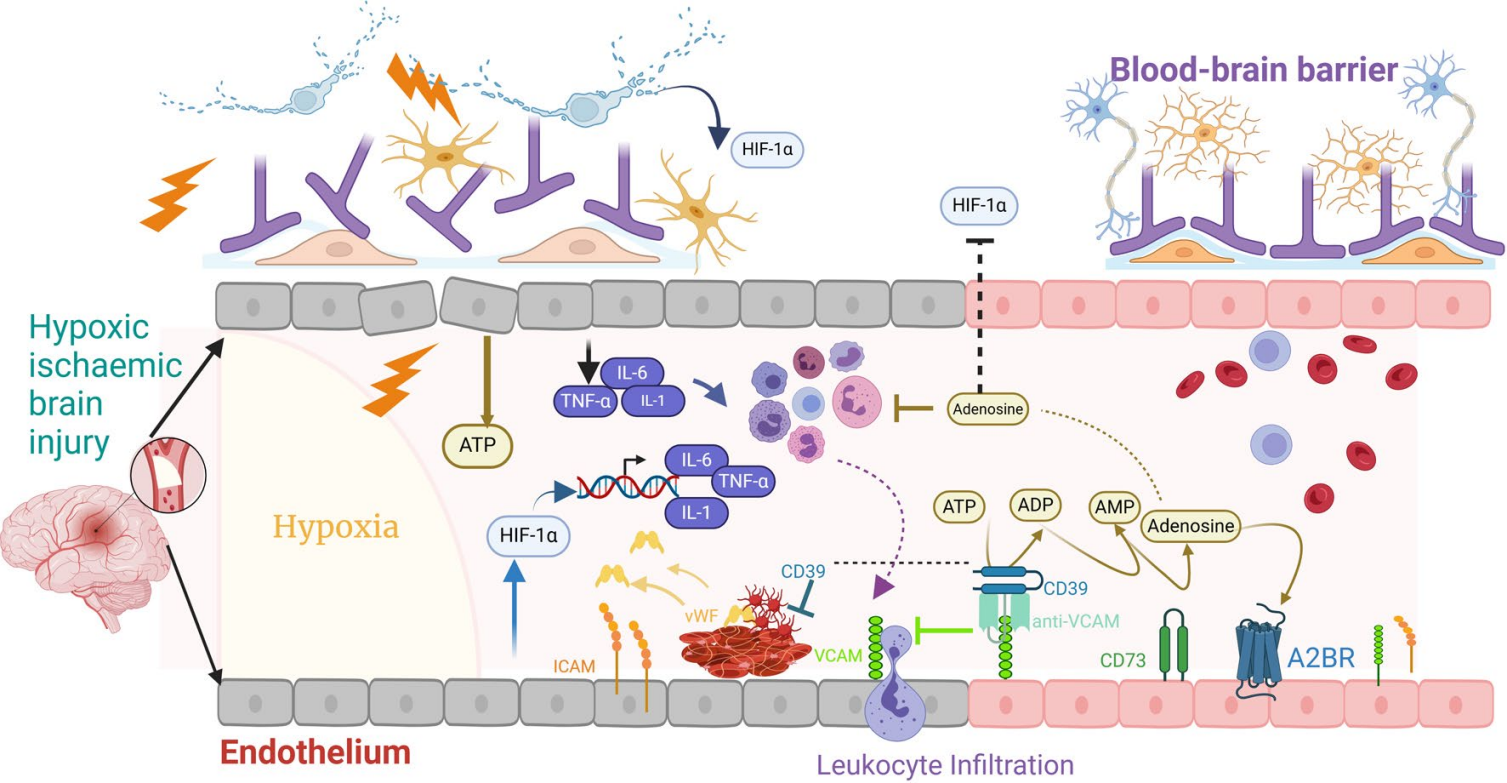
Postulated mechanism by which anti-VCAM-CD39 protects the brain against hypoxic-ischaemic brain injury. Hypoxia after cessation of blood flow followed by reperfusion activates endothelial cells, causing them to release inflammatory mediators including ATP and cytokines TNF-α, IL-1 and IL-6. TNF-α stimulates upregulation of VCAM-1, while hypoxia directly upregulates ICAM-1. This enhances adhesion and transmigration of monocytes into the brain parenchyma. vWF released from injured endothelial cells and present in the subendothelial matrix, provides a surface for platelet adhesion and subsequent platelet aggregation ultimately resulting in clot formation. Prolonged hypoxia increases the expression of pro-inflammatory cytokines through the activation of HIF-1α in neurons and endothelial cells. These processes culminate in further endothelial activation, blood-brain barrier disruption, microglial activation and cell death. Anti-VCAM-CD39 is a bifunctional molecule that blocks endothelial transmigration by blocking VCAM-1 and delivers recombinant CD39 to the site of endothelial activation. CD39 converts proinflammatory ATP to adenosine, which activates A2B receptors to downregulate HIF-1α. Created in https://BioRender.com ATP: adenosine triphosphate; HIF-1α: hypoxia inducible factor-1; ICAM-1: Intercellular adhesion molecule-1; TNF-α: tumour necrosis factor VCAM-1: Vascular cell adhesion molecule-1; VWF; (von Willebrand factor)

We showed that *anti-VCAM-CD39* promoted a targeted increase in adenosine generation, and via A_2B_ receptor signalling, reduced neuroinflammation and promoted endothelial protection; the postulated working mechanism is illustrated in Fig. 5. However, we acknowledge that additional work is needed to elucidate the exact mechanisms of protection by anti-VCAM-CD39. Interestingly, while it appears to be adenosine-driven in this model, it differs to that seen in the ischaemic stroke model, where much of the protection appears to be due to *anti-VCAM-CD39* mediated catabolism of eATP. eATP catabolism was likely reducing P2X 7 receptor signaling, microglial activation and NLRP3 inflammasome formation, thereby reducing neuroinflammation. However, in addition to ATP hydrolysis, anti-VCAM-CD39 caused an increase in microvascular perfusion, suggesting that it also sustains adenosine generation that protects the ischaemic penumbra in the setting of mouse focal ischaemic stroke [16]. The differences in mechanisms further highlights the importance of purinergic signalling in brain injury caused by ischaemia-reperfusion, and the need to further explore CD39-based therapeutics for these conditions.

### Methods

#### Mice

Experiments were performed in male C57BL/6 mice (age, 8 ± 0.2 weeks; weight, 28.2 ± 2 g) or CX3 chemokine receptor 1–green fluorescent protein positive (Cx3cr1gfp/+) mice expressing GFP under control of the Cx3cr1 promoter on a C57BL/6 background (age 4–6 weeks, weight 18–20 g) obtained from an in-house colony (Monash Animal Research Platform, Clayton Australia) where they were placed on a 12 h light-dark cycle with ad libitum access to food and water.

#### Dual carotid artery ligation (DCAL)

Transient global forebrain hypoxia (30 min) was induced using DCAL, as described previously [18]. Mice were anaesthetised in an induction box, with 5% Isoflurane and 0.8 L/min of oxygen. After the mouse was fully anaesthetised (identified by lack of movement, reflex and slowed breathing), the mouse was quickly transferred over to the surgery area adjacent to the induction area. The mouse was placed in the nose cone to maintain anaesthesia and positioned for surgery. Anaesthetic regimen during surgery is maintained between 1.5 and 2% Isoflurane and 0.7–0.8 L/min of oxygen. Surgery commenced once pedal reflex was not present. Core temperature was maintained between 36.5 and 37.5 °C, using a heatpad set at a temperature range of 37–39 °C. Animal temperature was regulated using a rectal probe, which automatically regulated heatpad temperature based on core body temperature.

For DCAL, briefly, the right carotid artery is exposed and a calibrated flow probe is placed around the artery to measure blood flow velocity. After 10 min, the left carotid artery is permanently sutured to block blood flow in the artery. This creates a lesion on the left side of the brain that is the site of permanent occlusion. The right carotid artery is then transiently clamped for a period of 30 min, cessation of blood flow is first confirmed, before reperfusion is triggered. This transient occlusion creates a lesion on the right side of the brain. For sham controls, the left CCA was permanently ligated, and the mice were allowed to recover.

#### Design of the *anti-VCAM-CD39* expression construct and protein production

Anti-VCAM-1 ScFv was generated by Prof Claudia Gottstein and genetically fused to CD39. The construct was prepared in the baculovirus expression vector pBacPAK9 and contained the IL-3 signal sequence, FLAG epitope and cDNAs for scFv VCAM-1 and for soluble non-targeted human CD39. Recombinant *anti-VCAM-CD39* was then produced using SF9 insect cells and purified by FLAG-Sepharose affinity-binding as published [16]. To fully evaluate the efficacy of the bifunctional aspect of *anti-VCAM-CD39*, we also created 2 control constructs. (1) Non-targeted CD39, which contained a scrambled ScFv sequence but retained CD39 activity measured via ATPase (adenosine) activity assay (described below). Non-targeted CD39 was also similarly expressed in SF21 cells followed by FLAG-affinity purification. CD39 activity of this control was 3 times less (not shown), therefore during administration, 3 times the dose was delivered (1.5 mg/kg). (2) The second control was *anti-VCAM-inactive CD39*, which was the full-length *anti-VCAM-CD39* construct that binds to VCAM-1 but the enzymatic activity of CD39 was abrogated by decreasing the pH to 5.5 for 2 min using 0.01 M HCl, before and then neutralising it with 0.05 M Tris HCl, pH 8.8.

VCAM-1 binding ELISA: 96-well plates (Costar) were coated with 0.25 µg of recombinant mouse VCAM (In Vitro Technologies Pty Ltd, 643-VM-050) or BSA resuspended in 50 µl of coating buffer (Carbonate Buffer Solution (0.05 M carbonate-bicarbonate buffer, pH 9.6), Sigma Australia) overnight at 4 °C. After washing with 1XPBS 0.05% tween (PBS-T), wells were blocked with 1% BSA in 1X PBS-T for 1 h at room temperature. BSA (negative control), anti-VCAM-CD39 or non-targeted CD39 (1 µg/well, 50 µl/well) were then added for 1 h at room temperature. After washing 1XPBS-T, anti hCD39 (Ancell # 188 − 040), 1:400 diluted in 1% BSA for 1 h at room temperature. After washing with 1X PBS-T, anti-mouse HRP (Polyclonal Rabbit Anti-Mouse Immunoglobulins/HRP, Dako, P0260) was added (1/5000 diluted in 1%BSA) for 1 h at room temperature. After washing, 100 µl TMB substrate solution (Invitrogen, N301) was added for 10 min after which 50µL stop solution was added (Invitrogen, SS03) to each well. OD450nm was measured with a plate reader (ClarioStar Plus, BMG Labtech Australia). Averaged, BSA blank subtracted values were graphed.

### Immunofluorescence

Frozen brain sections (10 μm) were dried in the dehumidifier dark box for roughly 30 min at room temperature. They were then washed twice in wash buffer (1x PBS/0.5% Tween20) for 5 min each. Auto-fluorescence block (0.1% Sudan Black in 80% ethanol) was done for 3 min at room temperature. The slides were washed once for 5 min in distilled H_2_O, followed by 2 5-minute washes in wash buffer. Protein block (1x PBS/4% BSA/10% normal horse serum (NHS)/2% Tween) was applied for 30 min at room temperature. The slides were then incubated with rabbit anti-VCAM-1 (1:200, EPR5047: Abcam, MA, USA), rabbit anti-ICAM-1 Monoclonal Antibody (1A29; Thermo Fisher Scientific Australia, 1:200), rat anti-mouse CD31 Antibody (1:400, Biolegend Australia) or sheep anti-von Willebrand Factor (vWF; 1:400) (ab11713: Abcam, MA, USA) in antibody dilution buffer (1x PBS/2% BSA/5% NHS/0.3% Tween) at 4 °C overnight.

The next day, the slides were washed for 3 × 10 min in PBS after which, Alexa Fluor^®^ 488 Donkey anti-Rabbit IgG (H + L), Donkey anti-Rat IgG 488 or Alexa Fluor 594 Donkey anti-Sheep IgG (H + L) (1:900; Thermo Fisher Australia) diluted in antibody dilution buffer was added onto the slides. The slides were then incubated in the dehumidifier dark box for 2 h at room temperature. Following this, the slides were again washed for 3 × 5 min in wash buffer, followed by a 2-minute wash in distilled H_2_O. Slides were incubated for 20 min in 10 µg/mL Hoechst 33,342 in PBS plus 0.1% Triton X-100 and then washed for 3 × 10 min in wash buffer and coverslipped, taking care to prevent bubble formation. The slides were left to cure for at least 30 min in the dark and stored in the dark until imaging. Images were captured using the Nikon TiE (Nikon, Tokyo, Japan; Monash Micro Imaging Platform) or VS200 research slide scanner (Olympus Australia; Monash Histology Platform).

### Anti-VCAM-CD39 binding

To confirm binding of anti-VCAM-CD39 to the endothelial cells, anti-VCAM-CD39 was labelled fluorescently with AF 546 as per manufacturer’s instructions using the Alexa Fluor 546 Protein Labelling Kit (Thermo Fisher Scientific Australia). Murine endothelial cells were treated with TNF-α (100 ng/ml) overnight and the fluorescently labelled AF 546 anti-VCAM-CD39 (1 µg/ml) was added. Cells were then co-stained with rabbit-anti-VCAM-1 antibody (1:50, Biolegend) and Hoechst (5 µg/ml, Thermo Fisher Scientific Australia) as nuclei marker, for 30 min at 37 °C. Cells were washed twice with media and imaged with confocal microscopy. 40x images with 2x optical zoom were captured using the Nikon A1R HD Galvano scanner (Monash Microimaging, Alfred Research Alliance). Images were analysed using Fiji (NIH Image J).

To study binding of anti-VCAM-CD39 in vivo, intravital imaging was performed using a published protocol [38] on mouse mesenteric veins using a custom-equipped Nikon A1R imaging system. We used terminally anesthetised male monocyte reporter Cx3cr1gfp/+ mice which revealed accumulation of Cx3cr1-GFP + monocytes within the injured endothelium. Fluorescently labelled antibodies targeting endothelial cells (Ms CD31-BV421; 2.5 µg, BD Biosciences Australia), VCAM-1 (Alexa Fluor^®^ 647 anti-mouse CD106 Antibody 2.5 µg, Biolegend, Australia), as well as labelled (Alexa Fluor 546) anti-VCAM-CD39 construct were administered via tail vein injection. A localized endothelial injury was inflicted on the vein using the galvanometric scanner with the 405 nm laser (200 mW, power = 80%, pixel dwell = 12 µs/pixel), and images acquired pre-injury and at 5 min post injury as described previously [38].

#### Treatment

We tested *anti-VCAM-CD39* at 0.125 mg/kg and 0.5 mg/kg and determined that the lowest therapeutic dose was 0.125 mg/kg, which we administered intravenously 3 h post-surgery. For non-targeted CD39, a direct comparison of ADPase activity was calculated by incubating sequential doses of the construct and anti-VCAM-CD39 in a malachite green assay as described [39]. The batch of non-targeted CD39, also produced in-house, was found to be approximately 3-times less active than the anti-VCAM-CD39 and therefore we injected a dose of 1.5 mg/kg of this agent. Anti-VCAM-inactive CD39 was administered at a dose of 0.5 mg/kg. Mice were assigned a random sample number, and all mouse groups were blinded during subsequent assessment. All animals were left to recover on the heat pad for at least 4 h after procedure. Animals were separated and given supportive treatment and left on the heat rack overnight [18].

#### Euthanasia and tissue harvesting

At 24 h post-surgery, mice were anaesthetized with Lethabarb (Pentobarbitone, 90 mg/kg, Australia), and transcardially perfused with phosphate buffered saline (PBS) pH 7.4. Unless otherwise stated, a 6 mm section of the infarcted brains was dissected and homogenised to 300 mg wet weight of tissue per 1 ml of Lysis Buffer (10mM Hepes pH 7.4, 10% Sucrose, 2mM EDTA, 0.1% CHAPS, 5mM DTT, 10ug/ml Aprotinin).

#### Functional assessment

Neurological assessment was done through Bederson scoring as described previously [18, 40] 20–24 h post-surgery whereby an independent blinded assessor scored mice based on their forelimb flexion, circling behaviour, and balance beam test.

#### Magnetic resonance imaging

At 24 h post-stroke, in-vivo MRI Imaging was performed using a 9.4 T/20 cm Bruker MRI with actively decoupled volume transmit and surface-receive coils as described previously [41]. Refer to supplementary methods for further information.

#### Caspase-3/7 activity assay

The fluorogenic substrate (N-Acetyl-Asp-Glu-Val-Asp-7-amido-4-trifluoromethylcoumarin (Ac-DEVD-AFC) was used to measure caspase-3/7 activity as previously described [18]. The injured (ipsilateral) and uninjured (contralateral) cortices were dissected and homogenized to 300 mg wet weight of tissue per ml of Caspase lysis Buffer (10mM HEPES pH 7.4, 10% Sucrose, 2mM EDTA, 0.1% CHAPS, 5mM DTT, 10 µg/ml Aprotinin). 10*µ*L of lysate was diluted in 90*µ*l of Caspase reaction Buffer (40mM HEPES pH 7.4, 200mM NaCl, 2mM EDTA, 0.2% CHAPS, 0.1% Sucrose, 3mM DTT) along with Fluorogenic Caspase-3 Substrate Ac-DEVD-AFC (Final concentration 13µM) in a black 96 well plate (Perkin Elmer, Australia). The reaction was allowed to take place at 37 °C and the fluorescence generated by the release of the fluorogenic group AFC on cleavage by caspase 3 was measured kinetically every 2 min for 200 min (Ex _400 nm_ and Em_505 nm_) using a BMG FLUOstar Omega Microplate Reader (BMG Labtech, Australia).

#### Blood brain barrier permeability assay

Albumin content in the brain was determined using the Mouse Albumin ELISA Quantitation Set (Bethyl Laboratories, USA) according to the manufacturer’s instructions and as described [18, 42]. Albumin in the brain parenchyma is reflective of the extent of BBB damage as published [42]. Total protein was quantitated using the BCA protein assay (Pierce). The amount of albumin (ng) per microgram protein in each sample was calculated from the standard curve. Albumin extravasation was calculated as difference in albumin in the ipsilateral and contralateral cortex for each animal for mice.

#### Determination of plasma ATP and adenosine levels

ATP and Adenosine levels in the plasma was determined as previously described [16] with a brief description as follows:

For measuring ATP levels, 25 µl of mice derived plasma was added to a 96-black well plate (#3904, Corning Australia) in duplicates together with ATP standard in triplicates (0.625nmol/L -10nmol/L including 0 nmol/L for blank) (#A7699, Adenosine 5’triphosphate disodium salt hydrate, Sigma Australia) to create a standard curve. Equal volumes of CellTiter-Glo^®^ Reagent (#G7570, Promega) were added in all wells and incubated after which luminescence was recorded on BMG FLUOstar Omega Microplate Reader (BMG Labtech, Australia) and ATP concentrations were calculated based on the standard curve generated.

For measurement of plasma adenosine, the Adenosine Assay kit was used (Fluorometric) (#ab211094, Abcam, Australia) according to the manufacturer’s instructions. Briefly, 5 µl of mice plasma was brought up to 50ul with the provided Adenosine Assay Buffer and equal volume of reaction mix was added into each standard and sample well. The plate was incubated at room temperature for 15 min in the dark and fluorescence was measured on FLUOstar OPTIMA plate reader at Ex/Em = 535/585nm.

#### Real-Time (RT)-PCR

Sections of ischemic brain tissue collected at 24 h post-DCAL were dissected and kept in RNAlater (Thermo Fisher Scientific, Australia) for 24 h before being stored at -80°c until use. For RNA extraction, tissues were homogenised in RLT lysis buffer (Qiagen, Hilden, Germany) and DNA-free RNA was prepared using RNeasy Mini Kit and DNase I set (Qiagen, Hilden, Germany) according to manufacturer’s protocols. Identical amounts of total RNA (1 µg per sample) were reverse transcribed to generate cDNA and real-time PCR was performed as previously described [18].

#### Measurement of cytokine levels in plasma

25µL of plasma from mice at 24 h post-DCAL was analysed in technical duplicates using the LEGENDplex™ Mouse Inflammation Panel (BioLegend, United States) according to manufacturer’s instructions.

**Western Blot**: Protein content in plasma was quantified using the bicinchoninic acid (BCA) assay (Thermo Scientific™ Pierce™, Australia). 2µL of plasma was subjected to SDS-PAGE (7.5% gel electrophoresed at 150 V for 1 h). The proteins were transferred onto PVDF membranes (Merck Millipore, Australia) at a current of 200 mA for 1 h as previously described. vWF levels in plasma were detected by sheep monoclonal anti-vWF (Abcam) (final concentration @ 0.437ng/mL) against a polyclonal donkey anti-sheep immunoglobulin (Thermo Fisher, Australia) (1:10000). The Bio-Rad Chemidoc MP Imaging system (Bio-Rad Laboratories Pty. Ltd. Australia) was used to obtain chemiluminescence images. These images were quantitated by densitometry using Image Lab™ software (Bio-Rad Laboratories Pty. Ltd. Australia). A standard curve was created using known concentrations of recombinant vWF (Abcam, Australia) assessed via western blot, and the amount of vWF was calculated from that curve.

#### Oxygen glucose deprivation (OGD) of cells

To simulate HIBI, confluent monocultures of immortalised mouse brain endothelial cells (bEND3; ATCC, BSL1) were subjected to OGD. Confluent bEND3 cells seeded on a 12-well plate (7.5 × 10^4^ cells per well) were washed once and equilibrated at 37 °C before cells were subjected to oxygen glucose deprivation (OGD) in Stimulation media (DMEM in 0.5%FBS) for 6 h before collection. *anti-VCAM-CD39* (10 µg/mL), ±adenosine receptor antagonist CGS-15,943 (200nM) (Sigma Aldrich, Australia), was then added. Next, cells were transferred to a hypoxic chamber (Modular Incubator Chamber; Billups-Rotheburg, Del Mar, CA, USA) which was flushed (6 min) with 100% nitrogen. The hypoxic chamber was kept humidified at 37 °C for the duration of the experiment (6 h). Anaerobic conditions were confirmed with the Anaerotest strip (Merck Millipore, Germany).

#### Cell death assay

Necrotic cell death was assessed by the lactate dehydrogenase (LDH) assay. Media and lysates of cells were collected and the LDH assay was run according to manufacturer protocol (Roche, Australia).

#### Statistical analysis

was performed using Prism 9 software (GraphPad, US). Normality tests were run to determine subsequent statistical test. Confirming normality, comparison of experimental datasets was performed by one-way ANOVA with Dunnett’s post-hoc correction or two-way ANOVA with Sidak’s or Dunnett’s post-hoc correction as stated. Non-normal datasets were compared with Kruskal-Wallis test with Dunn’s multiple comparisons test. Differences between two groups were assessed by two-tailed student t-tests (unpaired or unpaired with Welch’s correction for parametric data and Mann-Whitney test for non-parametric data). *P* < 0.05 was considered significant.

## Electronic supplementary material

Below is the link to the electronic supplementary material.

Supplementary Material 1

Supplementary Figure 1.4a

Supplementary Figure 1.4b

## Data Availability

No datasets were generated or analysed during the current study.

## Abbreviations

ADP: Adenosine diphosphate
ANOVA: Analysis of Variance
ATP: Adenosine triphosphate
BBB: Blood brain barrier
BCA: Bicinchoninic acid
bEND3: Brain endothelial cells
cDNA: Complementary DNA
Cx3cr1gfp/+: CX3 chemokine receptor 1–green fluorescent protein positive
DCAL: Double carotid artery ligation
DMEMORs: Dulbecco’s modified eagle mediumOdds ratios
DMSOPIR: Dimethyl sulfoxidePoverty income ratio
eATPPA: Extracellular adenosine triphosphate
HIBIRAR: Hypoxic ischaemic brain injuryRed blood cell distribution width to albumin ratio
HIF-1RCS: Hypoxia-inducible factorRestricted cubic spline
HRPRDW: Horseradish peroxidase
IRI: Ischaemia-reperfusion injury
IL: Interleukin
LDH: Lactate dehydrogenase
MRI: Magnetic resonance imaging
OGD: Oxygen glucose deprivation
PBS: Phosphate-buffered saline
PFA: Paraformaldehyde
RT-PCR: Real time polymerase chain reaction
ScFv: Single chain fragment variable
TBS: Tris-buffered saline
TNF-α: Tumour necrosis factor-α
VCAM-1: Vascular cell adhesion molecule 1
VLA-4: Very late antigen-4
vWF: Von willebrand factor
