# Supplementary material for: Endothelial -targeted CD39 is protective in a mouse model of global forebrain ischaemia
Source: J Neuroinflammation. 2025 Apr 21;22:115. doi: 10.1186/s12974-025-03394-7 (PMC12013200; doi:10.1186/s12974-025-03394-7)
Supplement: Supplementary file 1 — Supplementary Material 1 [file 12974_2025_3394_MOESM1_ESM.docx]

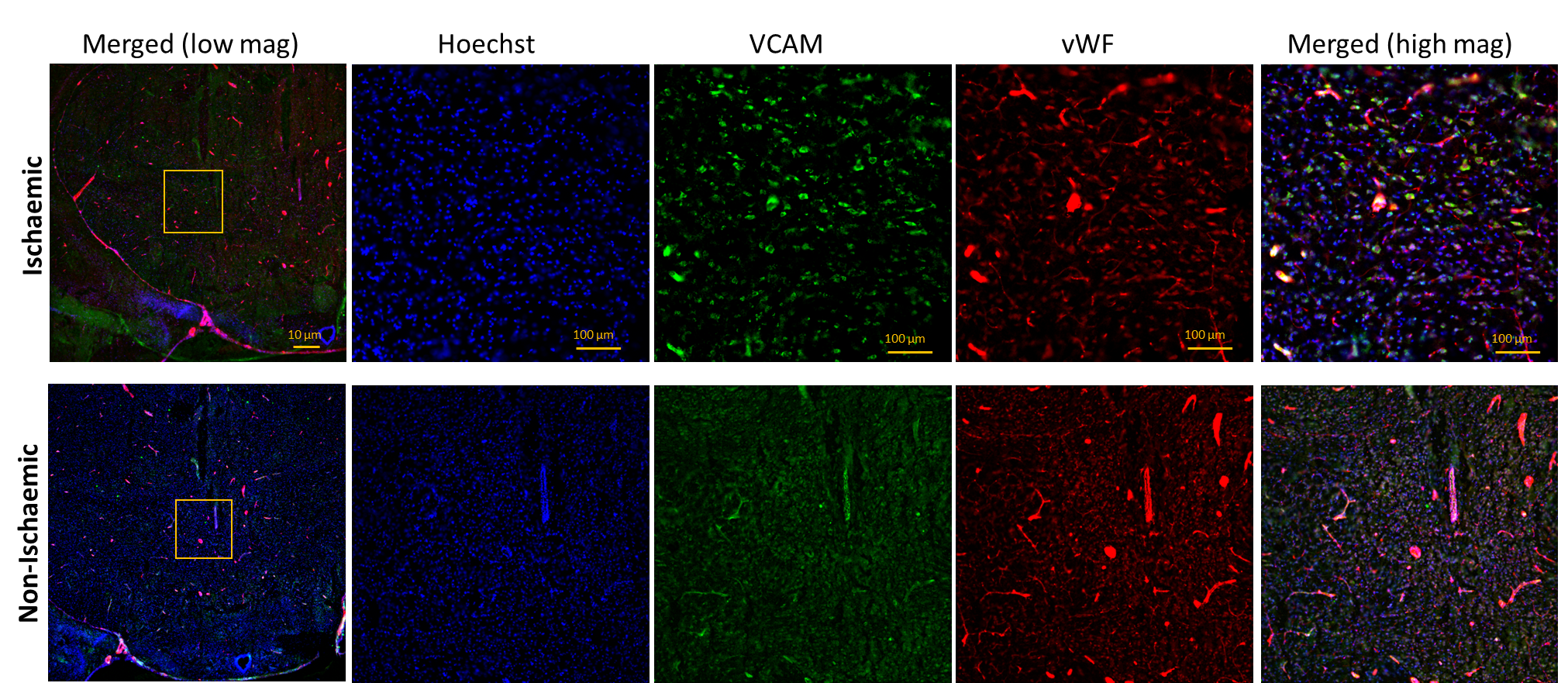


Supplementary Figure 1.1: Endothelial VCAM-1 is upregulated in the ischaemic area of the brain showing endothelial activation 3h post- DCAL induced global forebrain ischaemia. Brain sections have been stained with Hoechst (blue), anti-VCAM-1 (green) and anti-vWF (red) as an endothelial marker.

A)


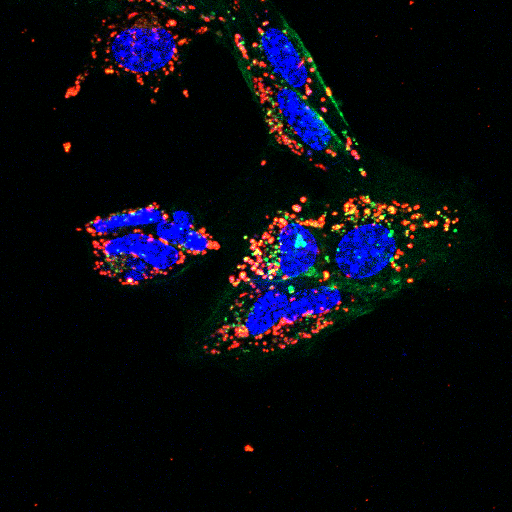

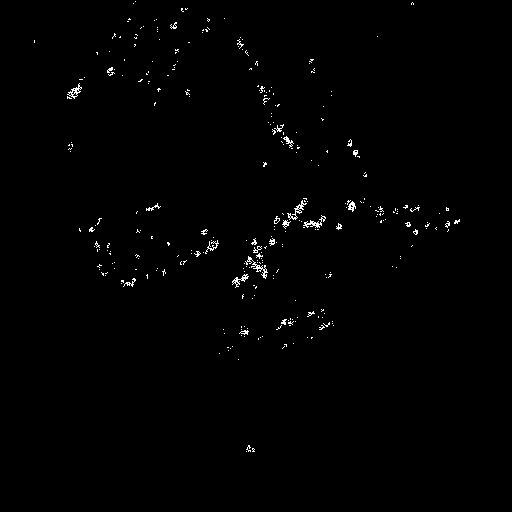

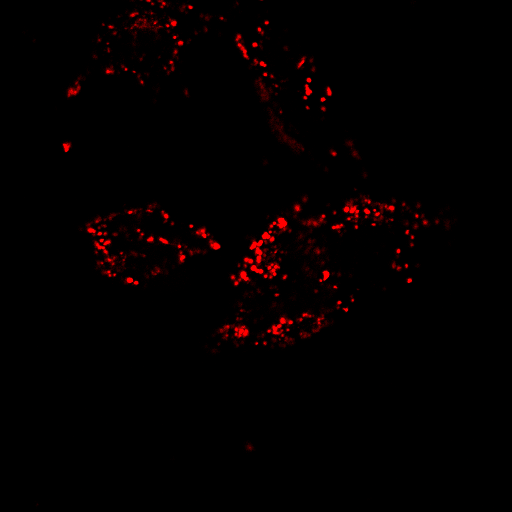

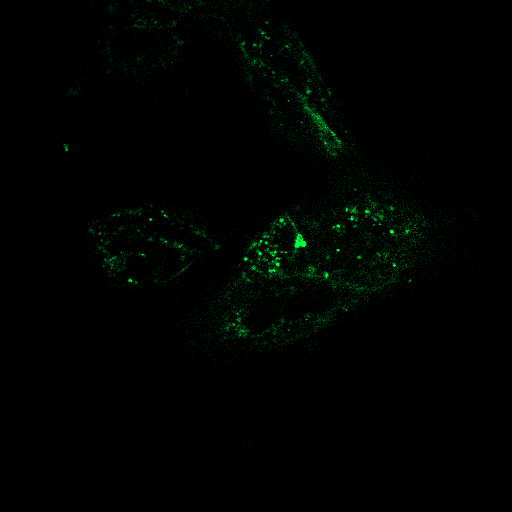


Anti-VCAM-CD39-AF 546

Anti-VCAM

Merged with Hoechst stain

Colocalisation matrix


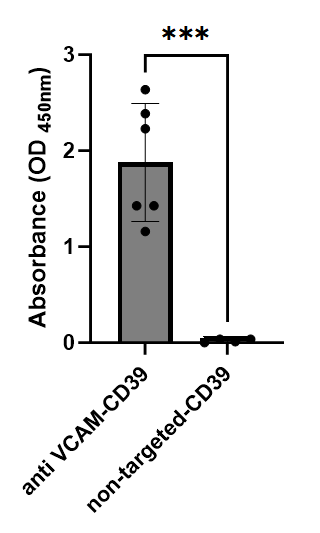


B)0

**Supplementary Figure 1.2.** Anti-VCAM-CD39 binds to VCAM-1 on (A) murine endothelial cells: In vitro: confocal microscopy shows that anti-VCAM-CD39-conjugated to Alexafluor 546 (red) binds to VCAM-1 (labelled with anti-VCAM-1 antibody; green) on endothelial cells stimulated overnight with 100 ng/ml TNF-α. The right panel shows co-localisation areas of anti-VCAM-CD39 with VCAM-1. (B) Non-targeted CD39 does not bind to VCAM-1 in an ELISA whereby 96-well plates are coated with recombinant mouse VCAM-1 and anti-VCAM-CD39 or non-targeted CD39 are then added and anti-human-CD39 is used as a probe (*** = p<0.001; n=6, unpaired t-test).


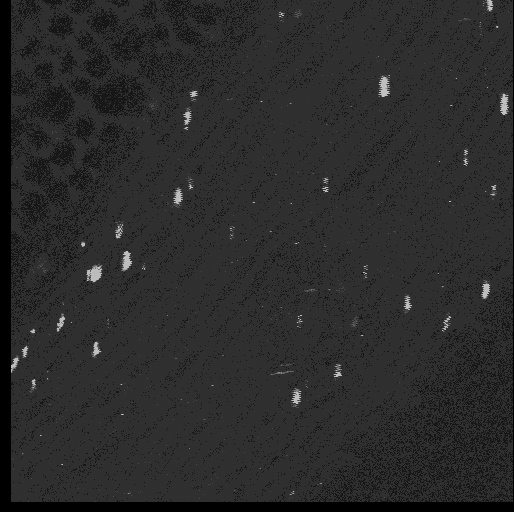

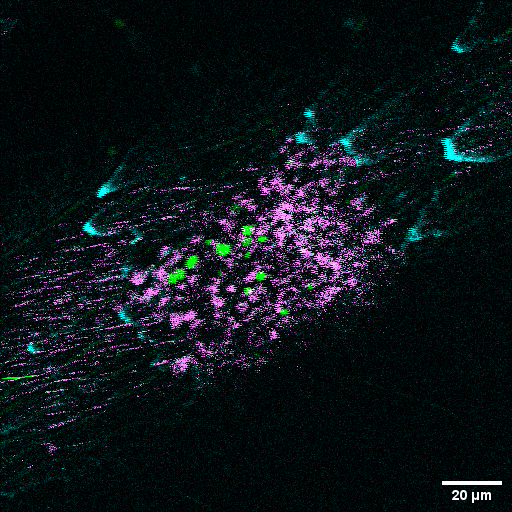


B i

B ii

**
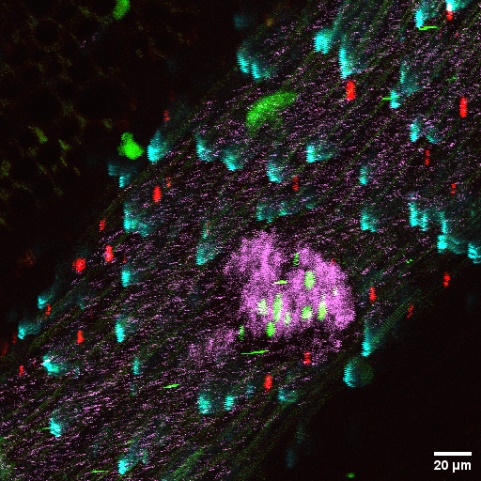
Supplementary Figure 1.3:** Anti-VCAM-CD39 binds to the vessel wall in vivo: Maximum intensity Z projections of a snapshot of confocal intravital imaging of the mesenteric vein following laser-induced endothelial injury in CX3CR1-GFP mice shows (A) prominent VCAM-1 upregulation (Alexa Fluor 647; pink) in the endothelium with abundant accumulation around the site of injury (BV421; stained with CD31- blue) within and surrounding the injured site with intravascular accumulation of monocytes (GFP; green) within the injured site. (B) (i) When anti-VCAM-CD39 labelled with Alexafluor 546 (red) is injected, it binds on the endothelial surface. (ii) Image J cololcalisation analysis between anti-VCAM-CD39-546 and CD31 showing colocalisation of the construct with endothelial cells.

A

Supplementary video 1.4: Video data corresponding to maximum intensity Z-project of (a) supplementary fig 1.3a (b) supplementary fig 1.3b

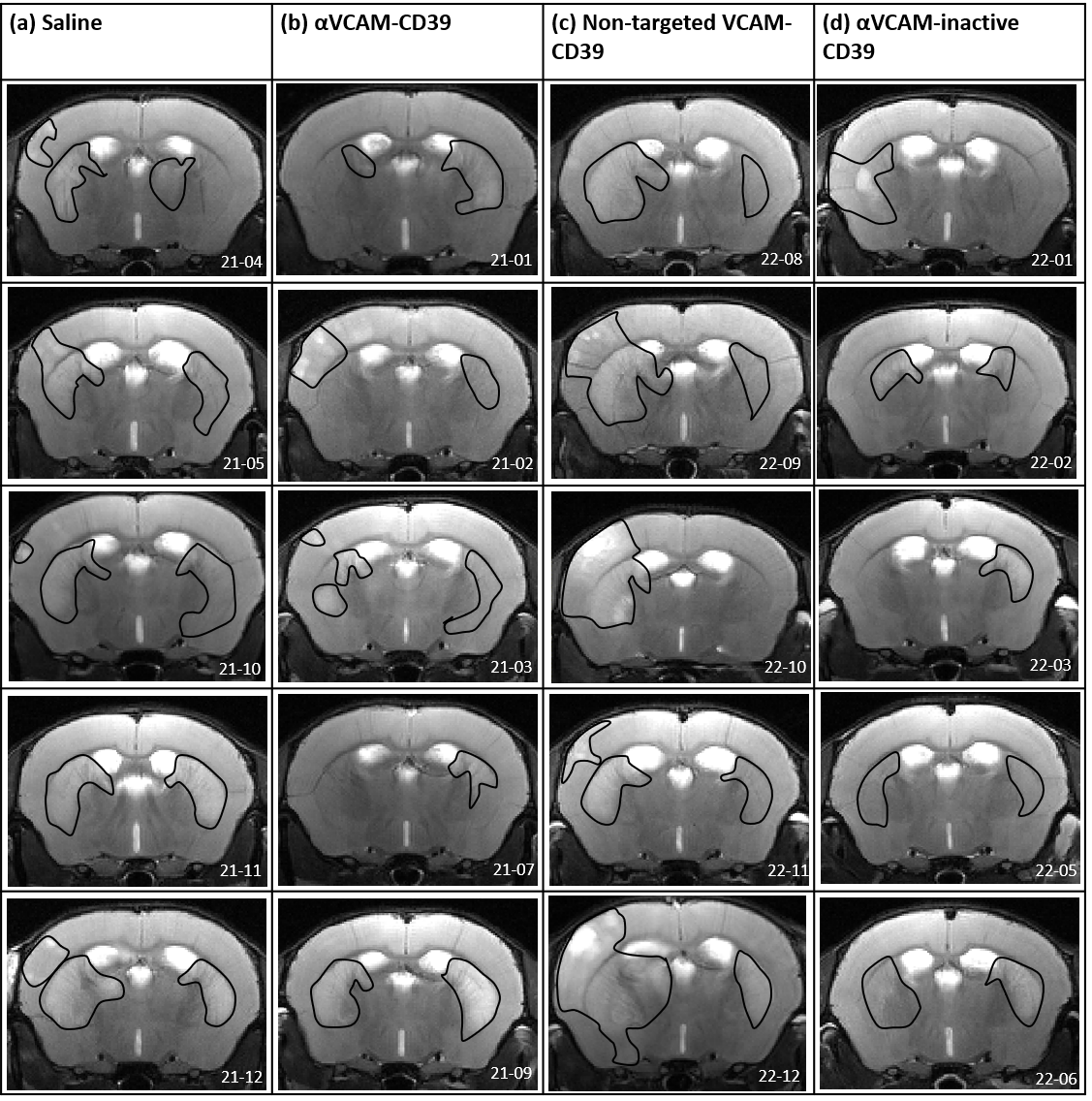


**Supplementary Figure 2.1:** MR T2* imaging of n=5 mouse brains post-DCAL after treatment with (a) saline, (b) αVCAM-CD39, (c) non-targeted VCAM-CD39, (d) anti-VCAM-inactive CD39 distinctly show reduced infarction following anti-VCAM-CD39 administration, consistent with the graph in Fig 2A.


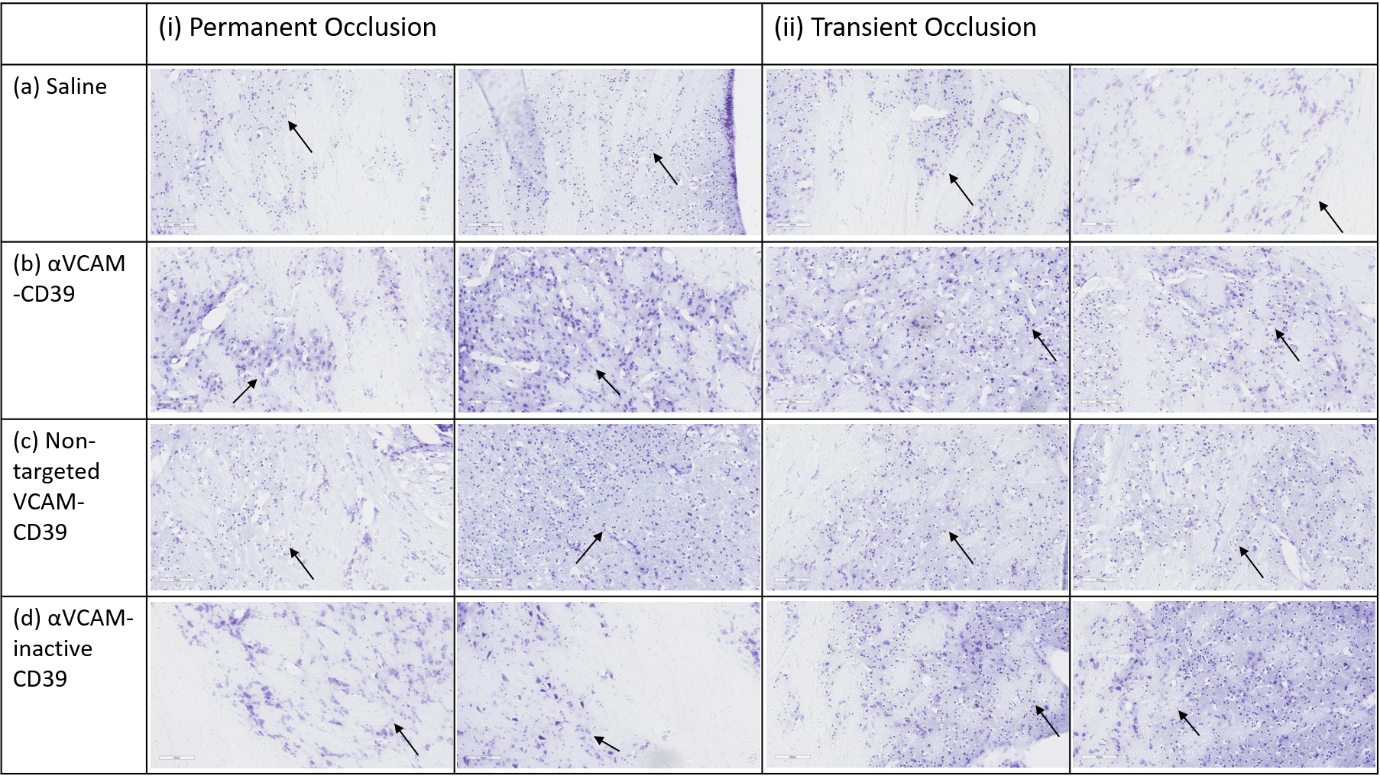
**Supplementary Figure 2.2:** Cresyl Violet staining of both (i) left and (ii) right brain hemispheres post-DCAL after treatment with (a) saline, (b) anti-VCAM-CD39, (c) non-targeted VCAM-CD39, (d) anti-VCAM-inactive CD39 at 20x magnification. This distinctly shows that the cells in both hemispheres after (ai and aii) saline treatment were of abnormal and degenerated morphology, with condensed or absent neuronal bodies. Such cell morphology was also seen after (c) non-targeted VCAM-CD39 and (d) anti-VCAM-inactive CD39 treatment. As expected, corresponding with reduced infarct size after (b) anti-VCAM-CD39 treatment, there was more normal cell morphology, and intact and round Nissl bodies were observed in both hemispheres.


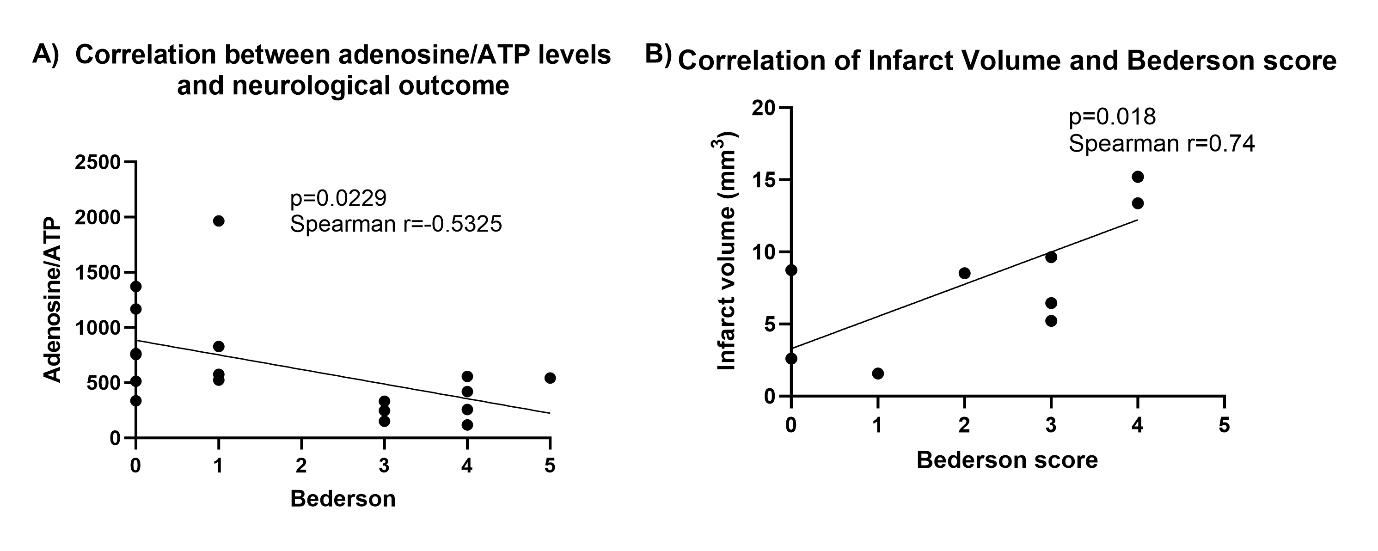


**Supplementary Figure 3:** ATP/adenosine levels inversely correlate with Bederson scores (n=18).


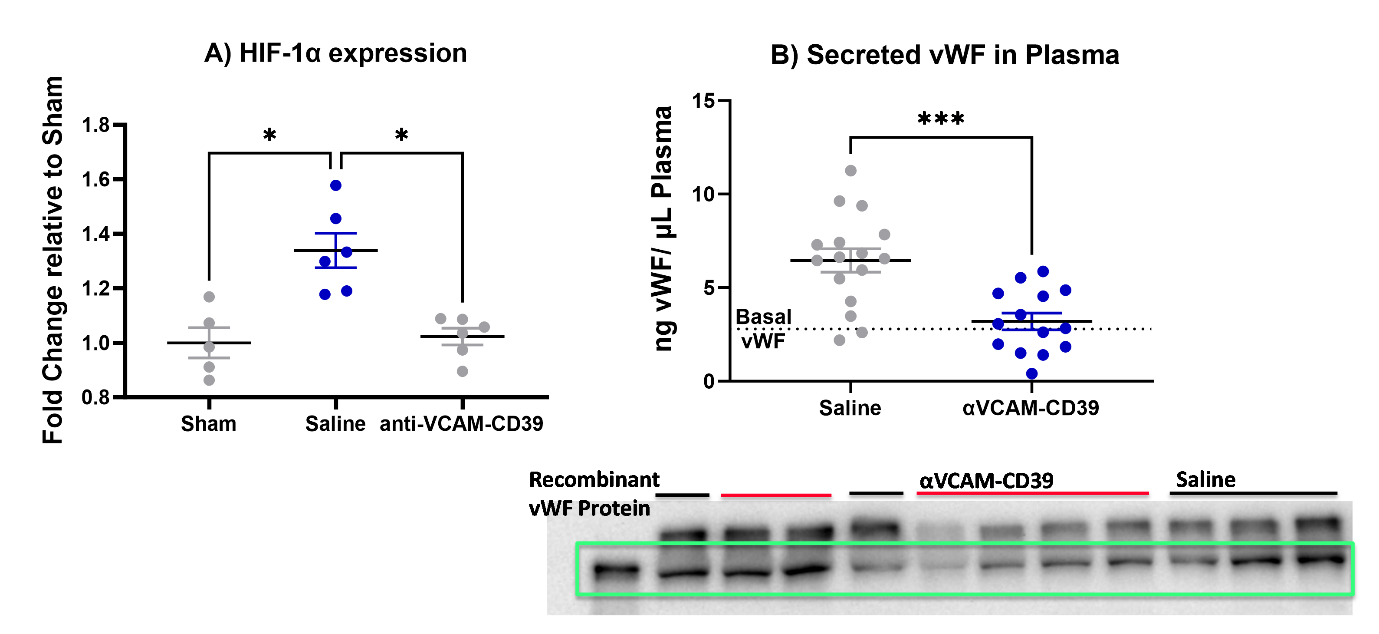


275 kDa


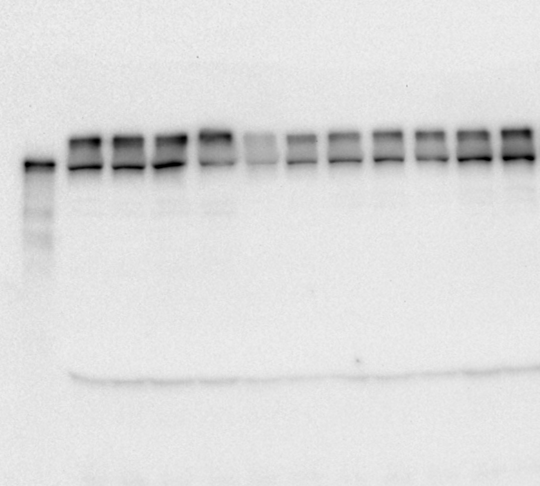


275 kDa

**Supplementary Figure 4:** Full western blot from figure 4 (Plasma vWF)
